# The C-Terminal Domain of Y-Box Binding Protein 1 Exhibits Structure-Specific Binding to Poly(ADP-Ribose), Which Regulates PARP1 Activity

**DOI:** 10.3389/fcell.2022.831741

**Published:** 2022-06-21

**Authors:** Konstantin N. Naumenko, Mariya V. Sukhanova, Loic Hamon, Tatyana A. Kurgina, Rashid O. Anarbaev, Aswin Mangerich, David Pastré, Olga I. Lavrik

**Affiliations:** ^1^ LBCE, Institute Chemical Biology and Fundamental Medicine (ICBFM), Novosibirsk, Russia; ^2^ SABNP, Univ Evry, INSERM U1204, Université Paris-Saclay, Evry, France; ^3^ Department of Natural Sciences, Novosibirsk State University, Novosibirsk, Russia; ^4^ Department of Biology, Molecular Toxicology Group, University of Konstanz, Konstanz, Germany

**Keywords:** Y-box binding protein 1, PARP1, trans-poly(ADP-ribosyl) ation, poly(ADP-ribose), disordered C-terminal domain

## Abstract

Y-box-binding protein 1 (YB-1) is a multifunctional protein involved in the regulation of gene expression. Recent studies showed that in addition to its role in the RNA and DNA metabolism, YB-1 is involved in the regulation of PARP1 activity, which catalyzes poly(ADP-ribose) [PAR] synthesis under genotoxic stress through auto-poly(ADP-ribosyl)ation or protein trans-poly(ADP-ribosyl)ation. Nonetheless, the exact mechanism by which YB-1 regulates PAR synthesis remains to be determined. YB-1 contains a disordered Ala/Pro-rich N-terminal domain, a cold shock domain, and an intrinsically disordered C-terminal domain (CTD) carrying four clusters of positively charged amino acid residues. Here, we examined the functional role of the disordered CTD of YB-1 in PAR binding and in the regulation of PARP1-driven PAR synthesis *in vitro*. We demonstrated that the rate of PARP1-dependent synthesis of PAR is higher in the presence of YB-1 and is tightly controlled by the interaction between YB-1 CTD and PAR. Moreover, YB-1 acts as an effective cofactor in the PAR synthesis catalyzed by the PARP1 point mutants that generate various PAR polymeric structures, namely, short hypo- or hyperbranched polymers. We showed that either a decrease in chain length or an increase in branching frequency of PAR affect its binding affinity for YB-1 and YB-1–mediated stimulation of PARP1 enzymatic activity. These results provide important insight into the mechanism underlying the regulation of PARP1 activity by PAR-binding proteins containing disordered regions with clusters of positively charged amino acid residues, suggesting that YB-1 CTD-like domains may be considered PAR “readers” just as other known PAR-binding modules.

## Introduction

YB-1 is a multifunctional RNA-binding protein mainly involved in RNA metabolism and other processes related to the maintenance of genome stability in animals (Mordovkina et al., 2020; Sangermano et al., 2020). Initially, YB-1 was identified as an RNA-binding protein implicated in the regulation of transcription and RNA metabolism (Eliseeva et al., 2011). YB-1 has mainly a cytoplasmic localization and is reported to associate with cytoplasmic ribonucleoprotein (mRNP) granules (Nekrasov et al., 2003; Skabkin et al., 2004). On the other hand, YB-1 displays a nuclear localization in aggressive types of cancer resistant to chemotherapy which influences the sensitivity of cancer cells to anticancer drugs and the efficiency of chemotherapy (Bargou et al., 1997; Shibahara et al., 2001; Matsumoto and Bay, 2005; Kosnopfel et al., 2014; Bates et al., 2020). In addition, the translocation of YB-1 from the cytoplasm to nucleus has been reported, mainly, upon treatment with a DNA-damaging drug; these data support the idea that YB-1 has nuclear-specific functions in cancer cells (Stein et al., 2001; Fujita et al., 2005; Sorokin et al., 2005). YB-1 actions in the cytoplasm are predominantly associated with mRNA metabolism (Budkina et al., 2020), while the nuclear function of YB-1—in addition to its described role as a transcription factor—remains to be elucidated (Sangermano et al., 2020). The participation of YB-1 in DNA repair was recently suggested because of its interactions with damaged DNA and several repair proteins identified in in vitro studies using recombinant proteins and cells (Hasegawa et al., 1991; Ise et al., 1999; Das et al., 2007; Gao et al., 2009; Fomina et al., 2015; Alemasova et al., 2016; Alemasova et al., 2017). A possible mechanism by which YB-1 is connected with DNA repair and genome stability could be its interaction with PARP1 (Alemasova et al., 2015), which is a key DNA repair–regulatory protein (Chaudhuri and Nussenzweig, 2017; Lavrik, 2020). PARP1 is a member of the ADP-ribosyltransferases diphtheria toxin-like family [ARTDs, also known as poly(ADP-ribose) polymerases (PARPs)], which catalyze the transfer of ADP-ribose units from NAD^+^ onto amino acid residues (aa) of target proteins resulting in their mono- or poly (ADP-ribosyl)ation (MARylation or PARylation) (Lüscher et al., 2021). PARP1 is well known primarily as a DNA base excision factor and a single-strand break repair factor that is recruited to DNA damage and forms DNA repair foci, facilitating the repair process (Hanzlikova et al., 2017; Polo et al., 2019; Lavrik, 2020). PARP1 activates upon binding to damaged DNA thereby resulting in the synthesis of a PAR and a covalent modification of itself and other nuclear proteins (Alemasova and Lavrik, 2019). PAR synthesis is considered a local signal at sites of DNA damage, which could attract proteins through noncovalent PAR binding or/and modulate a protein’s function *via* the covalent modification of the protein with PAR (d'Amours et al., 1999; Teloni et al., 2015). In recent years, some proteins modulating PARP1 enzymatic activity were identified (Ouararhni et al., 2006; Masaoka et al., 2012; Sun, X. et al., 2016; Gibbs-Seymour et al., 2016); thus, PARP1-interacting and PAR-binding partners are under intensive investigation (Dasovich et al., 2021; Kliza et al., 2021). In this context, YB-1 is an attractive candidate for a PARP1-interacting partner because YB-1 is a target for the covalent PARylation (trans-PARylation) by PARP1 and shows noncovalent binding to PAR *in vitro* (Alemasova et al., 2015; Alemasova et al., 2019). Both poly- and mono-(ADP-ribosyl)ation of YB-1 by PARP1, PARP2, PARP10, or PARP14 were revealed in proteomic studies of the ADP-ribosylome in various cell lines (Gagne et al., 2012; Carter-O’Connell et al., 2014; Carter-O’Connell et al., 2016; Zhen et al., 2017; Kalesh et al., 2019). Our previous research indicates that YB-1 modulates PARP1 activity and can be trans-PARylated *in vitro* (Alemasova et al., 2015; Alemasova et al., 2018; Naumenko et al., 2020). Besides, the regulation of PARP1 activity by YB-1 depends on the formation of PARP1–YB-1 complexes with damaged DNA and YB-1 interaction with PAR, which is accompanied by a decrease in the average PAR polymer size formed during PARP1 auto-PARylation (Naumenko et al., 2020). The effect of YB-1 on PARP1 activity appears to be related to the ability of this protein to bind both PAR and damaged DNA (Naumenko et al., 2020). YB-1 consists of a disordered alanine/proline-rich (AP) domain, a cold-shock domain (CSD), and a long disordered C-terminal domain (CTD) carrying clusters of negatively and positively charged residues ∼30 aa each (Eliseeva et al., 2011). Structurally, the CSD is similar to the oligonucleotide/oligosaccharide-binding fold (OB-fold), contains RNA-binding motifs RNP-1 and RNP-2, and participates in specific interaction with RNA and sequence-independent binding to single- or double-stranded DNA (Tanabe, Y., 2015, Ise, T 1999; Bouvet et al., 1995b). The CTD is involved in nonspecific nucleic-acid binding and seems to mainly stabilize the protein–nucleic acid interactions (Kloks et al., 2002; Kim et al., 2013). PAR is regarded as a nucleic-acid–like polymer and has variable chain length reaching 200 or more ADP-ribose units (Althaus and Richter, 1987; Alvarez-Gonzalez et al., 1987). Additionally, the PAR polymer has either linear or branched structure (Hayashi et al., 1983). In contrast to DNA (or RNA), PAR has a ribose-phosphate-phosphate-ribose backbone and contains two negative charges per nucleotide unit thus being more acidic than DNA or RNA (Alvarez-Gonzalez and Jacobson, 1987). RNA/DNA-binding proteins can directly interact with PAR, and PAR-binding modules in these proteins often overlap with DNA/RNA-binding domains, which can be structured or disordered (Teloni and Altmeyer, 2015). Accordingly, both the CSD and CTD of YB-1 are involved in DNA/RNA binding and may interact with PAR. Our previous studies have revealed that the CTD of YB-1 is implicated in the regulation of PARP1 activity, namely, a deletion of the CTD abrogates both YB-1–dependent stimulation of PARP1 and YB-1 trans-PARylation (Naumenko et al., 2020). These results suggest that the CTD is required for the YB-1 binding to PAR or formation of YB-1–PARP1–DNA complexes, thereby affecting the PARP1 activity.

In the present study, we tested the hypothesis of YB-1 binding to PAR and DNA *via* a charge-dependent mechanism. We demonstrated a strong correlation between the positive charge of the YB-1 CTD and its ability to bind PAR and damaged DNA and to be trans-PARylated. The data showed that the deletion of basic aa 230–324 in the CTD severely reduces YB-1 binding to PAR and has only a modest impact on binding affinity for DNA. The deletion of three positively charged amino acids clusters in the YB-1 CTD caused a loss of 1) YB-1 binding to PAR and to damaged DNA and 2) YB-1 trans-PARylation by PARP1. Furthermore, PAR structural features such as branching and chain length were found to influence noncovalent binding of YB-1 and to have a strong influence on YB-1 trans-PARylation. Thus, the CTD can be considered a specific PAR-interacting module that is capable of binding to a wide range of PAR polymer structures having various polymer lengths and frequencies of branching.

## Materials and Methods

### Chemicals

Radioactive [*α*-^32^P] ATP was produced in the Laboratory of Biotechnology at ICBFM (Siberian Branch of Russian Academy of Sciences [SB RAS], Novosibirsk, Russia). Oligodeoxynucleotides were synthesized by Biosset (Russia) and the Laboratory of Biomedical Chemistry at ICBFM (SB RAS, Novosibirsk, Russia). NAD^+^ and *β*-nicotinamide mononucleotide were purchased from Sigma-Aldrich (United States, catalog # 481911 and N3501, respectively), whereas reagents for buffer and electrophoresis components from Sigma-Aldrich, United States (Tris, catalog #T6791; BSA, catalog # A9418; EDTA, catalog #E5134; HEPES, catalog #H3375), PanReacAppliChem, Germany (acrylamide/bis-acrylamide, catalog # A1089/A3636; Urea catalog # A1049), Molecular Group (DTT, catalog # 19733320), Merk (NaCl, catalog # 106404).

### Plasmid Construction

Plasmid pET-3-1-YB-1 containing full length (FL) cDNA of rabbit *YB-1* is a generous gift from Drs. L.P. Ovchinnikov and D.A. Kretov (Institute of Protein Research RAS, Moscow, Russia). PCR products containing the full-length YB-1–coding sequence or a sequence encoding a truncated form of YB-1 (consisting of aa 1–184, 1–230, or 1–279) were cloned into the pLate-31 plasmid vector according to the recommended protocol (ThermoFisher, United States, catalog #K1271). The sequences of mutant genes were confirmed at the SB RAS Genomics Core Facility (ICBFM SB RAS, Novosibirsk, Russia).

Plasmid pET32a-hPARP-1-His is a kind gift from Dr. M. Satoh (Université Laval, Québec, Canada). Mutation Y986S, Y986H, or G972R within the *PARP1* coding sequence was generated by site-directed mutagenesis with Q5-polymerase (New England Biolabs, United States, catalog #M0491S). The sequences of mutant *PARP1* genes were confirmed at the SB RAS Genomics Core Facility.

### Protein Expression and Purification

Recombinant YB-1 and its mutants [YB-1 (Δ1), YB-1 (Δ1-2), or YB-1 (Δ1-2-3)] were overexpressed in *Escherichia coli* BL21 (DE3) and purified. YB-1 was purified by Ni-NTA affinity chromatography (GE Healthcare United States, catalog # GE17-5255-01), Mono-S chromatography (GE Healthcare, United States catalog # GE17-5168-01), and Superdex 16/600 chromatography (GE Healthcare, United States, catalog # GE28-9893-35) as described earlier (Alemasova et al., 2017). YB-1 mutants were purified by Ni-NTA and Mono-S chromatography.

Recombinant wild-type (wt) PARP1 and mutants PARP1^Y986S^, PARP1^Y986H^, and PARP1^G972R^ were overexpressed in *E. coli* Rosetta (DE3)pLysS (Novogen, catalog # 70956-3) and purified by Ni-NTA agarose (GE Healthcare United States, catalog # GE17-5255-01) affinity chromatography, HiTrap Heparin High Performance (GE Healthcare, United States, catalog # GE17-0407-01) affinity chromatography, and deoxyribonucleic acid−cellulose (single-stranded calf thymus DNA) (Sigma-Aldrich, United States, catalog #D8273) affinity chromatography as described previously (Sukhanova et al., 2004).

Yeast nicotinamide mononucleotide adenylyltransferase (NMNAT) was kindly provided by Dr. S.I. Shram (Institute of Molecular Genetic Russian Academy of Science, Moscow, Russia).

### DNA Substrates

A fluorescein (FAM)-labeled DNA duplex (Nick) was obtained by hybridization of 5′-FAM- CCG​CTA​TTT​CAA​CCC​TTT​GCA​GTC​CCA​GAA​GG-3′ with complementary oligonucleotides (5′-GGC​GAT​AAA​GTT​GGG-3′ and 5′-pAAACGTCAGGGTCTTCC-3′) in a 1.0:1.5:1.5 ratio. The oligonucleotide mixture was incubated for 5 min at 95°C and then slowly cooled to room temperature.

A damaged pBR322 plasmid (New England BioLabs, Catalog #N3033L) was prepared by heat and acid treatment in combination with apurinic/apyrimidinic endonuclease 1 (APE1)-catalyzed cleavage of apurinic/apyrimidinic sites (Sukhanova et al., 2004).

### Preparation of Protein-free poly(ADP-Ribose) [PAR]

[^32^P]NAD labeled on the adenylate phosphate was synthesized using [*α* ^32^P]ATP (3000 Ci/mmol) and *β*-nicotinamide mononucleotide in a reaction catalyzed by NMNAT as described elsewhere (Alemasova et al., 2015). [^32^P]-labeled PAR was synthesized in a 200 μL reaction mixture consisting of 50 mM Tris-HCl pH 8.0, 40 mM NaCl, 1 mM dithiothreitol (DTT), 5 mM MgCl_2_, 100 nM Nick, 200 nM PARP1 (or 500 nM PARP1^Y986S^, PARP1^Y986H^, or PARP1^G972R^), and 10 μM [^32^P]NAD^+^ (20 μCi). The mixture was incubated at 37°C for 30 min. After that, DNA was removed by DNase I (New England BioLabs, Catalog #M0303L) treatment. The poly (ADP-ribosyl)ated PARP1 was incubated with 0.1 M NaOH at 37°C for 40 min, and then pH was adjusted to 7.5 with 0.1 M HCl. PAR was isolated from the resulting sample by phenol:chloroform:isoamyl alcohol (25:24:1) (Sigma-Aldrich, catalog #P2069) extraction and purified by ethanol precipitation. Resultant PAR samples were analyzed by electrophoresis in a denaturing urea 20% polyacrylamide gel with subsequent phosphorimaging. [^32^P]-labeled–PAR concentration was estimated as the amount of monomeric ADP-ribose incorporated into a polymer. [^32^P]NAD^+^ signal intensity (arbitrary units, a.u.) was used as a standard. The amounts of [^32^P]PAR produced were calculated by acquiring the signals from PAR resolved by the urea polyacrylamide gel electrophoresis (PAGE).

### A Radioactive Assay of PARP1 Activity and Protein Trans- and Auto-PARylation

To calculate the initial rates of PAR synthesis in the reaction catalyzed by PARP1 (or its mutants) in the presence or absence of YB-1 full length (FL) (or its mutants), the kinetics of [^32^P]-labeled–ADP-ribose incorporation into an acid-insoluble precipitate were assayed with [^32^P]NAD^+^ as a substrate. The reaction mixtures consisted of 50 mM Tris-HCl pH 8.0, 40 mM NaCl, 1 mM DTT, 100 μg/ml BSA, 10 mM EDTA, 100 nM PARP1 wt [or PARP1^Y986S^, PARP1^Y986H^, or PARP1^G972R^], 0.5–2.5 µM YB-1(FL) or 2.0 µM mutant YB-1, and 0.5 OD_260_/ml DNase I–activated calf thymus DNA (DNA_act_). The reaction components were mixed on ice. The reactions were initiated by the addition of NAD^+^ (0.4 μCi [^32^P]NAD^+^) to a final concentration of 20 μM. The mixtures were incubated at 30°C, 5 µl aliquots were taken at 1, 3, 5 and 10 min. The reactions were stopped by placing aliquots dropwise on Whatman 1 paper filters preimpregnated with 10% trichloroacetic acid (TCA). The PARylated proteins were precipitated on filters in the presence of TCA. To remove unreacted NAD^+^, the filters were washed four times with 5% TCA, then TCA was removed by means of 96% ethanol, and the filters were dried. [^32^P]-labeled–ADP-ribose incorporation into the acid-insoluble material (PARylated proteins) was quantified by radioautography using Typhoon FLA 7000 (GE Healthcare, United States). The data were plotted (*p* versus t) and fitted by logistic Equation 1:

$$\left[P\right]=\left[P_{max\;}\right]\cdot \left(1-e^{-kt}\right)\;$$ (1)

$$v=\frac{\;d\left[P\right]}{dt}=k\cdot \left[P_{max}\right]\cdot \;e^{-kt}\;$$ (2)

$$v_{0}={\frac{\;d\left[P\right]}{dt}|}_{t=0}=k\cdot \left[P_{max}\right]\;$$ (3)

where *p* is product PARP1-(ADP-ribose)n, *P*_*max*_ is maximum synthesis product concentration at t_∞_ *, t* is time, k is first-order rate constant. Calculated kinetic parameters, [P_max_] and k, can be used to calculate the initial rate of the reaction (Equation 3).

The protein PARylation assay was performed in reaction mixtures composed of 50 mM Tris-HCl pH 8.0, 40 mM NaCl, mM DTT, 100 μg/ml BSA, 10 mM EDTA, 100 nM PARP1 wt (or PARP1^Y986S^, PARP1^Y986H^, or PARP1^G972R^), 0.5–2.5 µM YB-1(FL) [or 2 μM YB-1 (Δ1), YB-1 (Δ1-2), or YB-1 (Δ1-2-3)], and 0.5 OD_260_/ml activated DNA. The reactions were initiated by the addition of NAD^+^ (0.4 μCi [^32^P]NAD^+^) to a final concentration of 20 μM and were allowed to proceed at 30°C for 10 min. The reactions were stopped by the addition of SDS sample loading buffer and heating for 1.5 min at 97°C and were analyzed by denaturing SDS-PAGE as described elsewhere (Laemmli, 1970). Bands of proteins labeled with [^32^P]ADP-ribose were visualized and quantified by phosphorimaging on Typhoon FLA 7000 (GE Healthcare, United States) and in the Quantity One Basic software.

### Evaluation of Half-Maximal Effective Concentrations (EC_50_) of YB-1–PAR Complexes

Complexes of YB-1(FL) or one of its mutants with PAR were subjected to an electrophoretic mobility shift assay (EMSA). The reaction was carried out in a mixture composed of 50 mM Tris-HCl pH 8.0, 40 mM NaCl, 1 mM DTT, 100 μg/ml BSA, 60 nM [^32^P]PAR (estimated by the calculation of the [^32^P]ADP-ribose amount incorporated into the polymer), and various concentrations of YB-1(FL) or its mutants. The reaction mixtures were incubated at 37°C for 5 min. Loading buffer consisting of 20% glycerol and 0.015% bromophenol blue was then added to the samples. Nondenaturing PAGE in a 5% gel (acrylamide/bis-acrylamide at 37.5:1) was performed for the analysis of complexes PAR–YB-1(FL) and PAR-YB-1 (Δ1), 10% PAGE (acrylamide/bis-acrylamide at 75:1) for the analysis of complexes PAR–YB-1 (Δ1-2) and PAR–YB-1 (Δ1-2-3), and 10% PAGE (acrylamide/bis-acrylamide at 75:1) for the analysis of complexes PAR^Y986S^–YB-1(FL), PAR^Y986H^–YB-1(FL), and PAR^G972R^–YB-1(FL) in TBE buffer at 4°C followed by phosphorimaging on a Typhoon FLA 9500 Biomolecular Imager (GE Healthcare). Bound- and unbound-PAR signals were quantified in the Quantity One Basic software. The data were fitted to an equation using the SigmaPlot software.

### Fluorescence Anisotropy Measurements of the Binding of YB-1 or its Mutants to DNA

The anisotropy measurements were performed at 25°C on a CLARIOstar multifunctional microplate reader and in the MARS Data Analysis Software (BMG LABTECH GmbH, Germany). Excitation wavelength was 482 nm (the 482-16 filter plus dichroic filter LP504), and emission wavelength was 530 nm (530-40 filter). The binding reactions were conducted in Corning black 384-well polystyrene assay plates. Reaction mixtures consisting of a buffer (50 mM Tris-HCl pH 8.0, 40 mM NaCl, 1 mM DTT, and 100 μg/ml BSA), 0–6000 nM YB-1(FL) [or YB-1 (Δ1), YB-1 (Δ1-2), or 0–20000 nM YB-1 (Δ1-2-3)], and 50 nM FAM-labeled Nick were prepared on ice.

The data were plotted (F versus C) and fitted to a four-parameter logistic equation:

$$F=F_{0}+\left(F_{max}\;-\;F_{0}\right)÷\left[1+\;{\left[\frac{EC_{50}}{C}\right]}^{n}\right]$$

where F is the measured anisotropy (mA) of a solution containing the FAM-labeled DNA at a given concentration (C) of YB-1 protein, F_0_ represents anisotropy of a solution of the labeled DNA alone, F_max_ is anisotropy of the DNA saturated with YB-1, EC_50_ denotes the concentration of protein at which F = (F_max_–F_0_)/2, and n is the Hill coefficient.

### Dynamic Light Scattering Measurement of the Size Distribution of PARylated PARP1 and its Mutants

These measurements were performed to determine the hydrodynamic radius (R_h_) of PARylated molecules. All DLS measurements were conducted as described previously (Vasil’eva et al., 2019). PARylation reactions were carried out directly in a quartz cuvette used for DLS measurements. The reaction mixture (25 μL) was composed of 25 mM HEPES-NaOH pH 7.5, 100 mM NaCl, 1 mM DTT, 10 mM MgCl_2_, 2.5 μM Nick, and 2.5 μM PARP1 wt (or PARP1^Y986S^, PARP1^Y986H^ or PARP1^G972R^). Samples were equilibrated for 1 min and then the auto-PARylation reactions were initiated by the addition of NAD^+^ to a final concentration of 1 mM. R_h_ measurement was performed every 3 min after the PARylation reaction initiation. After 40-min incubation, the reaction was stopped by the addition of EDTA to a final concentration of 10 mM, and R_h_ was measured in the EDTA-treated sample.

R_h_ of the particles was calculated *via* the Stokes–Einstein equation under the assumption of the spherical shape of the PARylated molecules:

$$R_{h}=\frac{k\cdot T}{6\cdot \pi \cdot \eta \cdot D}$$

where D is the diffusion coefficient determined by DLS, k denotes Boltzmann’s constant, T is absolute temperature, and η represents solvent viscosity assumed here to be the viscosity of water containing buffer components at 25°C.

### Atomic Force Microscopy Experiments and Image Analysis

For experiments with the auto-PARylation of PARP1 wt or its mutants (PARP1^Y986S^ and PARP1^Y986H^), 40 nM PARP1 was incubated with 10 ng/μL pBR plasmid in a buffer (50 mM Tris-HCl pH 8.0, 25 mM NaCl, 10 mM MgCl_2_, and 1 mM DTT). The reactions were initiated by the addition of NAD^+^ to a final concentration of 0.25 mM followed by incubation for 1 h at 37°C. Next, the samples were diluted 10-fold with AFM deposition buffer (12.5 mM HEPES-NaOH pH 8.0, 12.5 mM KCl, and 1 mM DTT) and immediately deposited on a mica surface. For AFM imaging, the samples were processed as described before (Sukhanova et al., 2016). After that, the mica surface was rinsed with a 0.02% uranyl acetate solution, rapidly rinsed with pure water (Millipore), and air-dried before AFM imaging in ambient air (Révet et al., 1998) by means of Nanoscope V Multimode 8 (Bruker, Santa-Barbara, CA, United States) in peakforce tapping (PFT) mode with Scanasyst-Air probes (Bruker). In this experiment, continuous force–distance curves were recorded at 2048 × 2048 pixels and a line rate of 1.5 Hz, and the tip was oscillated in the vertical direction with an amplitude of 100–300 nm at low frequency (1–2 kHz).

## Results

### Truncation of C-Terminal Domain of YB-1 Impairs PAR Binding

**FIGURE 1:**
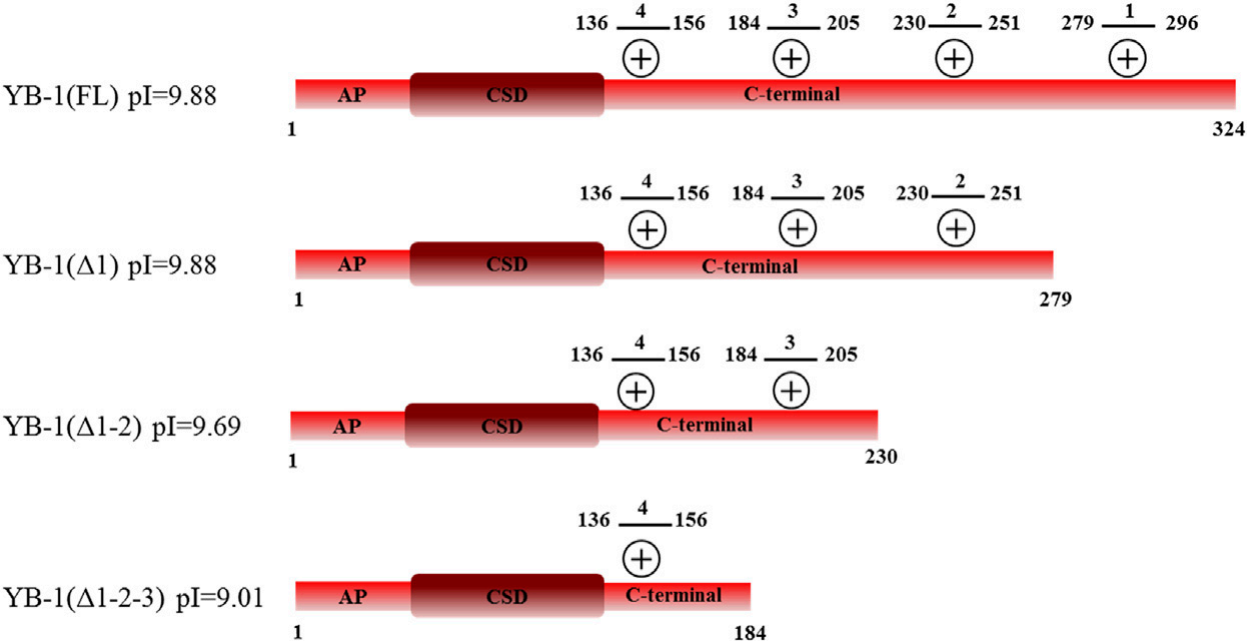
Domain structure of YB-1(FL) and its C-terminal deletion mutants: YB-1 (Δ1), YB-1 (Δ1-2), and YB-1 (Δ1-2-3). Designations: alanine/proline-rich (AP) domain, cold-shock domain (CSD), C-terminal domain (CTD) carrying four clusters (1_279-296_, 2_230-251_, 3_184-205_, 4_136-156_) of positively charged residues (+).

**FIGURE 2:**
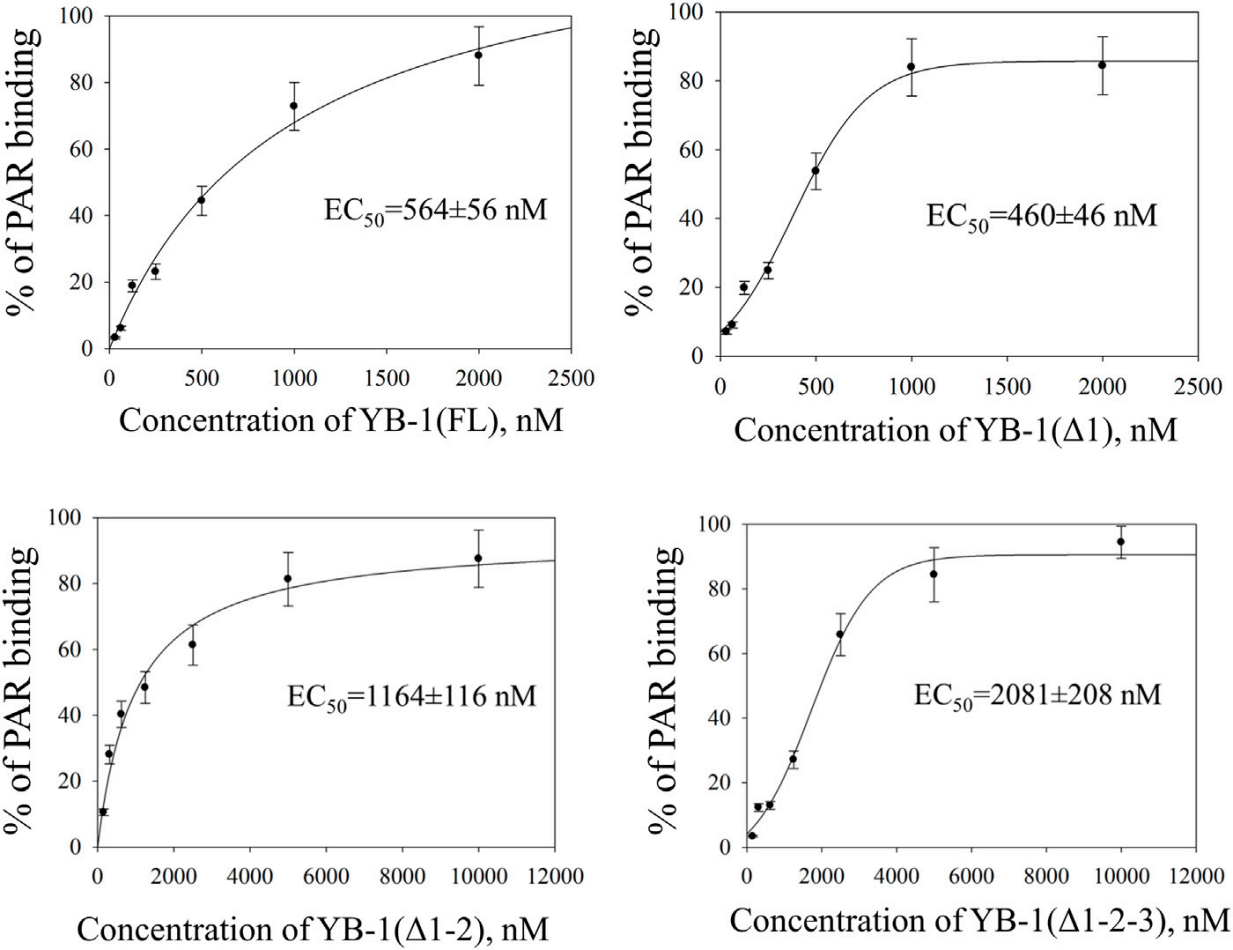
CTD shortening weakens YB-1 affinity for PAR. Graphs show quantification of EMSA data (Supplementary Figure S1) and represent the mean values of three independent experiments with error bars (±SD). The binding parameters (EC_50_) of YB-1 interaction with PAR was determined as YB-1 concentration resulting in 50% substrate binding. EC_50_ values are the mean (±SD) of three independent experiments. The reaction mixtures contained 60 nM [^32^P]-labeled PAR and YB-1(FL) or its C-terminal deletion mutants at the indicated concentrations.

Two possible mechanisms have been proposed to explain YB-1–dependent regulation of PARP1 activity: {1} formation of a heterotrimeric PARP1–YB-1–damaged DNA complex, where YB-1 is a predominant target of PARylation, and {2} formation of a YB-1 complex with PAR covalently attached to PARP, where again YB-1 is the main target of the modification (Naumenko et al., 2020). In both cases, the interaction of YB-1 with damaged DNA or PAR influences the regulation of PARylation reactions. Previously, we showed that the CTD is essential for the control of PARP1 activity by YB-1 *in vitro*, whereas the AP-CSD fragment has only a minor influence on PARP1 activity (Naumenko et al., 2020). Therefore, we hypothesized here that efficient formation of a YB-1–PAR or YB-1–damaged DNA complex depends on the positively charged clusters within the CTD. The CTD has been reported to play an important part in the regulation of YB-1 interaction with DNA and RNA, regulation of YB-1 protein–protein interactions, and control over the formation of YB-1 multimers (Tafuri and Wolffe, 1992; Murray, 1994; Bouvet et al., 1995a; Pisarev et al., 2002; Kretov et al., 2019). Nevertheless, general involvement of the CTD in PAR binding is not understood completely. The disordered YB-1 CTD, aa 130–324, is the largest domain of YB-1 which contains four clusters of positively charged residues (aa 136–156, 184–205, 230–251, and 279–296; Figure 1). We propose that through electrostatic interactions, positively charged amino acids can contribute to the YB-1 binding to DNA and PAR. Therefore, deletion of these clusters should influence the formation of stable complexes between YB-1 and PAR (or DNA) and reduce the efficiency of YB-1 interaction with these molecules. To assess the contribution of the CTD positively charged clusters to the binding of YB-1 to PAR and DNA, truncated mutants of YB-1 were prepared in this work. These mutants contain deletions of one [YB-1 (Δ1)], two [YB-1 (Δ1-2)], or three [YB-1 (Δ1-2-3)] positively charged clusters in the YB-1 CTD (Figure 1). Characteristics of the binding of YB-1(FL) or its deletion mutants to a protein-free PAR polymer were compared by the EMSA (Figure 2; Supplementary Figure S1: Gel shift).

There was no significant difference in the PAR binding affinity only in the comparison between the YB-1 (Δ1) mutant and YB-1(FL). By contrast, the deletion of two or three positively charged clusters in the C terminus dramatically reduced the ability of YB-1 to bind PAR (Figure 2). EC_50_ values of interactions YB-1(FL)–PAR and YB-1 (Δ1)–PAR were two-to four-fold lower (∼500 nM) than those of YB-1 (Δ1-2)–PAR (1164 nM) and YB-1 (Δ1-2-3)–PAR (2081 nM). The data suggested that a deletion of at least two positively charged clusters (aa 231–304) in the YB-1 CTD significantly reduces its binding to PAR.

**FIGURE 3:**
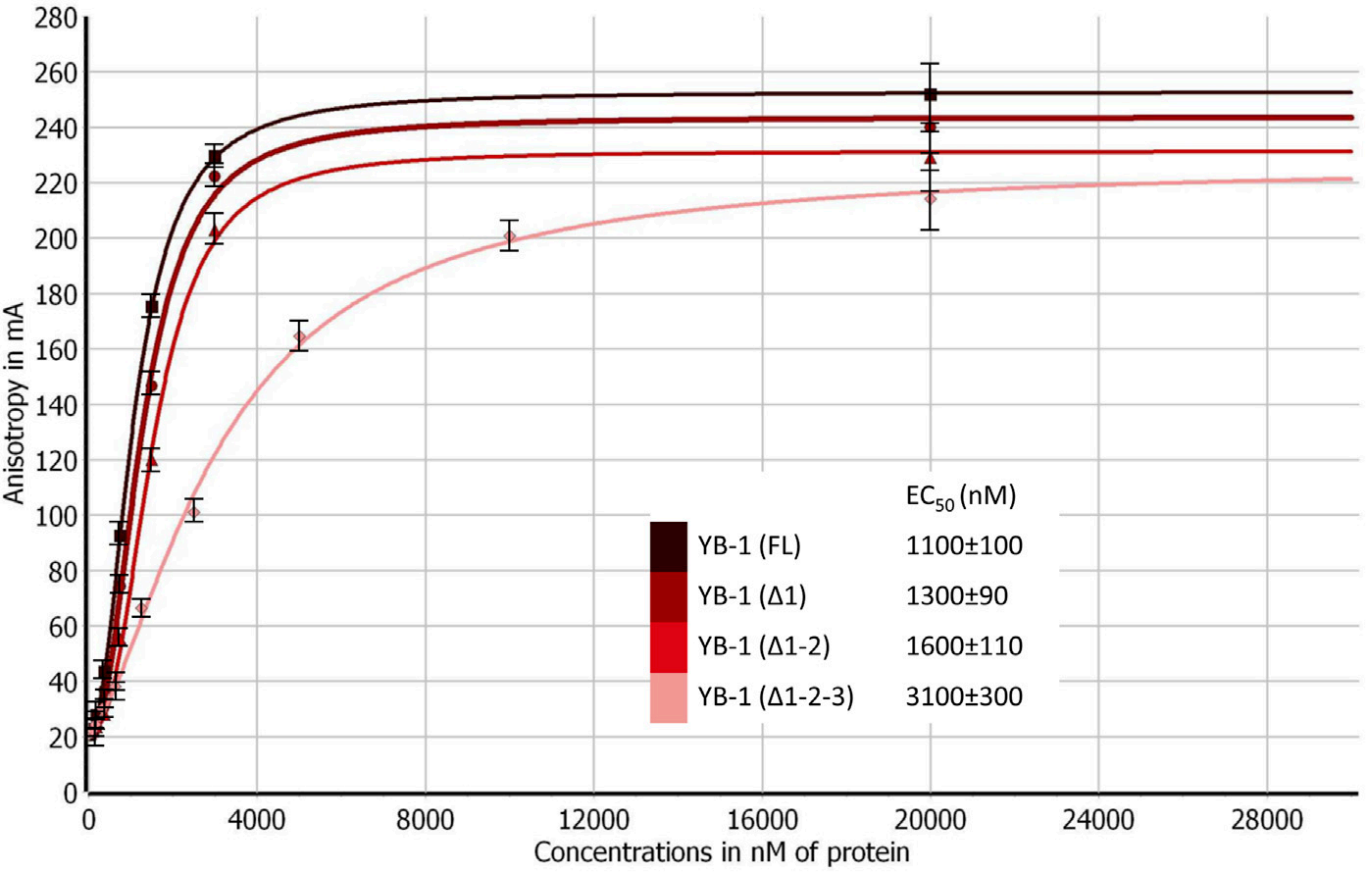
CTD shortening weakens YB-1 affinity for damaged DNA. Titrations of FAM-labeled DNA with YB-1(FL) or one of its deletion mutants. The reaction mixtures contained 50 nM FAM-labeled DNA duplex and either YB-1(FL) or one of its C-terminal deletion mutants as indicated. Graphs represent the mean values of three independent experiments with error bars representing ± SD. EC_50_ values are the mean (±SD) of at least three independent experiments.

Truncations of the CTD can also weaken YB-1’s DNA-binding affinity (Tanabe et al., 2015). To address this point, the efficiency of the binding of YB-1(FL) or its deletion mutants to DNA was measured by fluorescence anisotropy measurements (Figure 3). The gradual truncation of the CTD affected the YB-1–DNA interactions by weakening YB-1 binding affinity for DNA. Thus, YB-1 and its mutants bind to DNA with EC_50_ values ranging from 1100 to 3100 nM, and the YB-1 (∆1-2-3) mutant possesses ∼3-fold weaker affinity for DNA than YB-1 (FL) does.

These data meant that the CTD truncation in YB-1 correlates with attenuation of PAR-binding affinity; in addition, the shortest mutant, YB-1 (∆1-2-3), also has a much weaker DNA-binding ability in comparison with YB-1 (FL). Moreover, a deletion of two positively charged clusters in the CTD drastically impaired YB-1 binding to PAR, although YB-1 (∆1-2) still possesses a DNA-binding ability similar to that of the full-length protein.

### Truncation of C-Terminal Domain of YB-1 Decreases the Level of Trans-PARylation of YB-1

**FIGURE 4:**
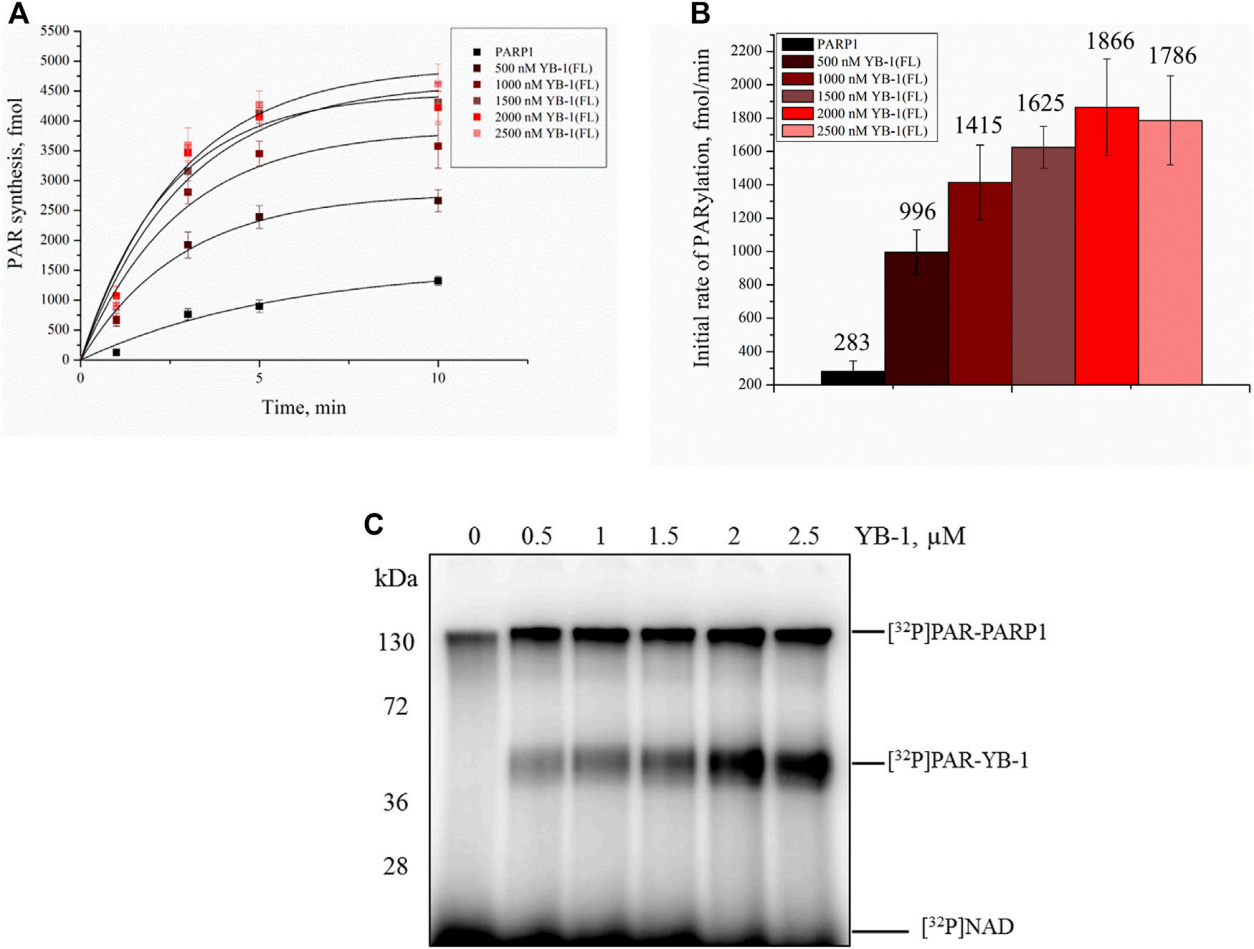
YB-1 increases the rate of PAR synthesis by PARP-1 and the PARylation level of protein targets (PARP-1 and YB-1). **(A)** Kinetics of PAR synthesis catalyzed by PARP1 in the absence or presence of various concentrations of YB-1(FL). Graphs represent the mean values of three independent experiments with error bars representing ± SD. **(B)** Initial rates of PAR synthesis estimated in the presence of various concentrations of YB-1(FL). PARP1 at 100 nM was incubated with 0.5 OD_260_/ml DNA_act_, 20 μM NAD^+^, and [^32^P]NAD (0.4 μCi) in the presence of 0.5–2.5 µM YB-1(FL) as indicated. [^32^P]PAR-modified proteins were precipitated with TCA and quantified radiographically. The initial rates (fmol/min) were determined from a direct analysis of progressive curve **(A)** which yields P_max_ and k (Equations 1 and 3), and represent the mean ± SD of three independent measurements. **(C)** PARP1 auto-PARylation and YB-1 trans-PARylation detected by SDS-PAGE and subsequent phosphorimaging. The reaction mixtures were composed of 100 nM PARP1, 0.5 OD_260_/ml DNA_act_, 0.5–2.5 µM YB-1 (or its mutant as indicated), 20 μM NAD^+^, and [^32^P]NAD (0.4 μCi).

**FIGURE 5:**
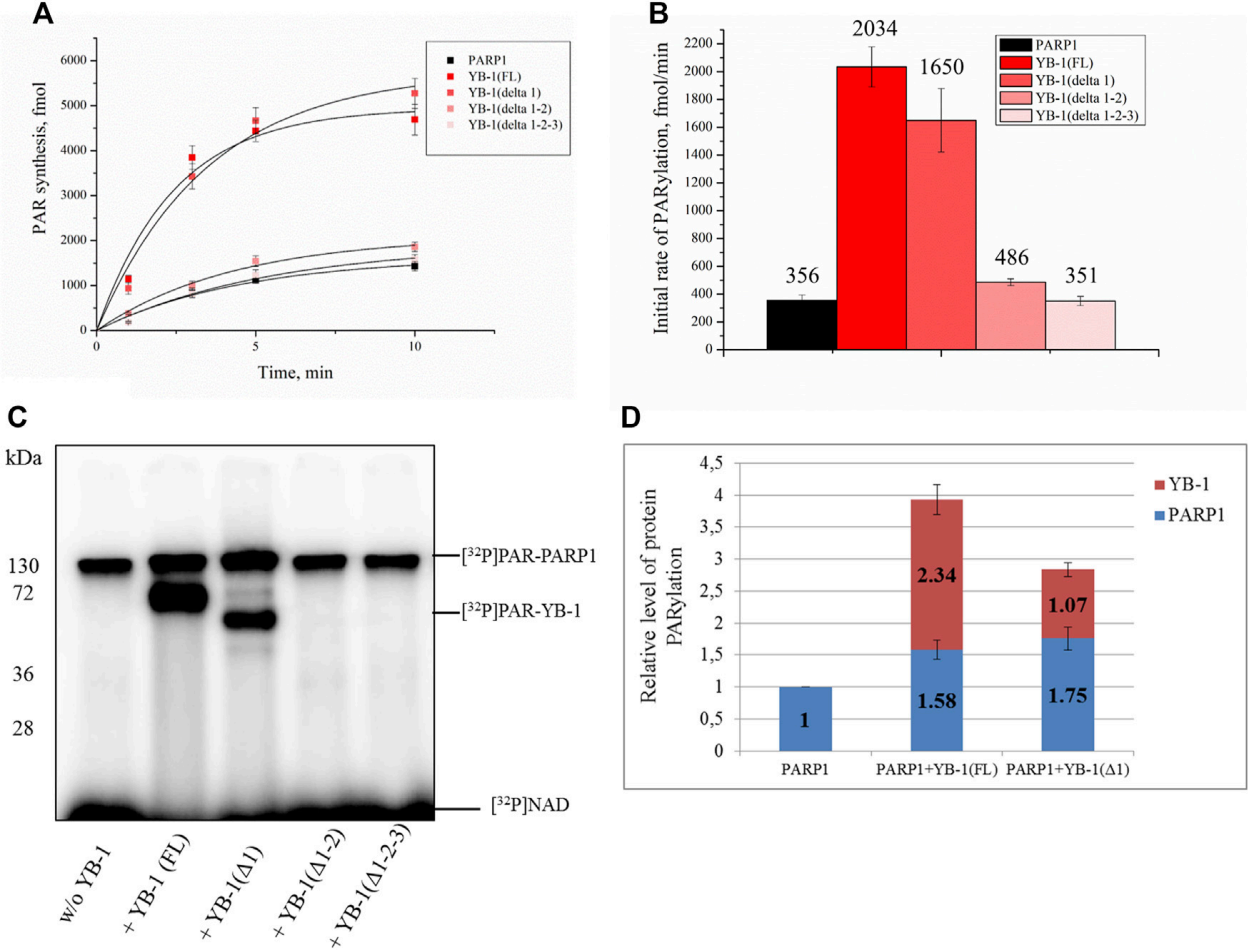
A positively charged region of the YB-1 CTD is required for the stimulation of PARP1 activity and YB-1 trans-PARylation. **(A)** Kinetics of the PAR synthesis catalyzed by PARP1 in the absence or presence of YB-1(FL) or its deletion mutants. Graphs represent the mean values of three independent experiments with error bars representing ± SD. **(B)** The initial rates of PAR synthesis estimated in the presence of either YB-1(FL) or one of its deletion mutants. PARP1 at 100 nM was incubated with 0.5 OD_260_/ml DNA_act_, 20 μM NAD^+^, and [^32^P]NAD (0.4 μCi) in the presence of 2.0 µM YB-1(FL), YB-1 (Δ1), YB-1 (Δ1-2), or YB-1 (Δ1-2-3) as indicated. [^32^P]PAR-modified proteins were precipitated with TCA and quantified radiographically. The initial rates (fmol/min) were determined from a direct analysis of progressive curve **(A)** which yields P_max_ and k (Equations 1 and 3), and represent the mean ± SD of three independent measurements. **(C)** PARP1 auto-PARylation and trans-PARylation of YB-1 mutants detected after SDS-PAGE with phosphorimaging. The reaction mixtures contained 100 nM PARP1, 2 μM YB-1(FL) or its mutant as indicated, 20 μM NAD^+^ [^32^P]NAD (0.4 μCi), and 0.5 OD_260_/ml DNA_act_. **(D)** The diagram presents relative levels of PARP1 auto-PARylation and YB-1 trans-PARylation (the mean ± SD of three independent experiments). The relative protein PARylation levels were normalized to the auto-PARylation data on PARP1 alone.

Our previous studies have shown that YB-1 stimulates the PAR synthesis catalyzed by PARP1 (Carter-O’Connell et al., 2018; Naumenko et al., 2020). To determine whether our data on YB-1 binding to PAR or DNA are consistent with the influence of YB-1 on PARP1 activity, we analyzed this activity in the presence of either YB-1(FL) or one of its deletion mutants: YB-1 (Δ1), YB-1 (Δ1-2), or YB-1 (Δ1-2-3) (Figures 4, 5). First, we estimated PARP1 activity and protein PARylation at various concentrations of YB-1(FL) (Figure 4). A higher concentration of YB-1 caused ∼ 6.5-fold acceleration of the initial rate of PAR synthesis, accompanied by an increase in both PARP1 auto-PARylation and YB-1 trans-modification (Figures 4B,C). These results are consistent with our previous data (Chen et al., 2018; Naumenko et al., 2020).

Next, we tested whether C-terminally truncated forms of YB-1 affect the PARylation reactions catalyzed by PARP1, namely the rates of PAR synthesis and protein PARylation (Figure 5). The stimulatory effect of YB-1 on PARP1 activity diminished with gradual shortening of the CTD in YB-1 (Figure 5B). Additionally, YB-1 mutants with truncated CTD yielded substantially lower levels of trans-PARylation (Figures 5C,D).

Although the presence of YB-1 (Δ1-2) caused only moderate stimulation of PARP1 activity and this mutant yielded only a low level of trans-PARylation, the YB-1 (Δ1-2-3) mutant had no noticeable effect on PARP1 activity and manifested no trans-PARylation (Figures 5B,C).

Consequently, the partial CTD deletion in YB-1 affects the interaction of YB-1 with both PAR and DNA and attenuates the stimulatory effect of YB-1 on overall PAR synthesis and own trans-PARylation. In particular, the YB-1 (Δ1-2-3) mutant has a weak affinity for PAR and DNA (Figures 2, 3) and fails to stimulate PARP1 activity. This mutant cannot be trans-PARylated either (Figure 5). This finding confirms the importance of the positively charged region of the CTD for PAR and DNA binding by YB-1 and for the regulation of PARylation reactions.

Together with our previous results showing that PARP1 regulation with YB-1 depends on the presence of DNA and formation of ternary complex of PARP1-DNA-YB-1 (Naumenko et al., 2020), these data provide further support of significant contribution of YB-1 interaction with PAR in regulation of PARP1 activity.

### The Affinity of YB-1 for PAR Depends on the Length and Frequency of Branching of the Polymer

**FIGURE 6:**
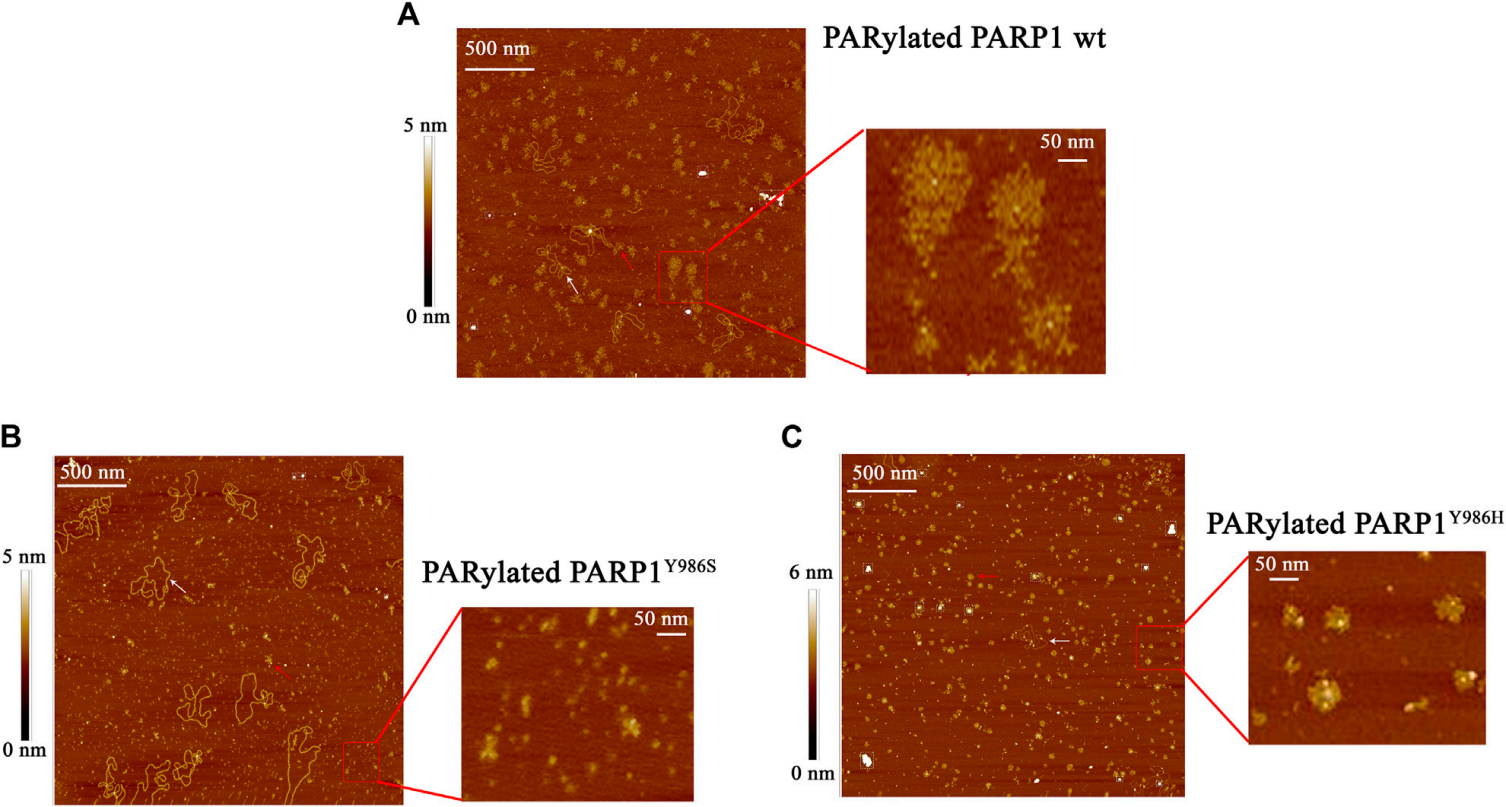
AFM visualization of the shape of PAR polymers synthesized by PARP1 wt **(A)** or its mutants: PARP1^Y986H^ **(B)** or PARP1^Y986S^ **(C)**. The large scale of AFM images illustrate auto-PARylation of PARP1 and its mutants, red rectangle shows the higher magnification image of modified molecules. White arrows indicate plasmid DNA molecules, and red arrows point to PARylated proteins. Scale bar: 500 nm; Z scale: 5 nm (for PARP1 wt and PARP1^Y986S^) and 6 nm (for PARP1^Y986H^).

Our data indicated that the stimulation of PARP1 activity by YB-1 is affected by the removal of three positively charged clusters in the CTD; for instance, YB-1 (Δ1-2-3) showed weaker binding affinity for PAR (Figure 2) and its trans-PARylation was not detectable (Figure 5). This result suggested that YB-1–PAR interactions play a pivotal part in the regulation of PARP1 by YB-1 and in YB-1 trans-PARylation reactions. PAR is a nucleic-acid–like polymer composed of ADP-ribose monomers, but in contrast to DNA or RNA, this polymer is known to have branched structures with branching points occurring every 20–50 ADP-ribose units, and its monomers have a twice higher negative charge (Althaus and Richter, 1987). Both the branching frequency of PAR polymers and chain length affect noncovalent protein binding and PAR polymer stability *in vitro* as well as PAR-dependent protein localization and reorganization on nuclear structures in the cell (Panzeter et al., 1992; Fahrer et al., 2007; Fahrer et al., 2010; Aberle et al., 2020; Rudolph et al., 2021). Furthermore, PAR branching frequency is reported to vary during different phases of the DNA damage–induced PARylation reaction, implying biological relevance of PAR structure (Aberle et al., 2020). Taking into account that the YB-1–PAR noncovalent interactions *via* the CTD make YB-1 a target for trans-PARylation (Figures 2, 5), the different chain length and branching frequency of PAR could regulate YB-1–PARP1 functional interactions. To clarify the influence of PAR structure on YB-1–dependent regulation of PARP1 activity, we used single-point mutants of PARP1 that synthesize short (PARP1^Y986S^), short hyperbranched (PARP1^Y986H^), or short hypobranched (PARP1^G972R^) PAR polymers (Rolli et al., 1997). Previously, the characteristics of PAR polymers produced by these PARP1 mutants have been determined by PAGE, two-dimensional thin-layer chromatography, HPLC and UPLC-MS/MS analysis (Rolli et al., 1997; Aberle et al., 2020). To test the enzymatic activity of the PARP1 mutants, we performed a PAGE-based assay of PARPs’ auto-PARylation and of PAR polymers (Supplementary Figure S2). Additionally, AFM was carried out to analyze morphological features of the auto-PARylated proteins [PARP1 wt, PARP1^Y986S^, and PARP1^Y986H^; Figure 6]. AFM imaging of PARP1 wt or its mutant (PARP1^Y986S^, PARP1^Y986H^, or PARP1^G972R^) after incubation with a DNA substrate in the presence of NAD^+^ was conducted next. PARP1’s and its mutants’ activation that was detected at the single-molecule level allowed us to analyze the morphology of the PAR polymers synthesized by these proteins (Figure 6). We noticed that these point mutations of PARP1 are accompanied by alterations in the shape and size of auto-PARylated PARP1 molecules detected by AFM. In general, the wild-type PARylated PARP1 molecules were larger than the mutant proteins’ molecules. This finding is in agreement with biochemical data indicating that all these mutations of PARP1 lead to synthesis of shorter chain length of PAR compared with the wild type (Rolli et al., 1997; Aberle et al., 2020). The AFM images revealed that PARylated PARP1 wt has star-shaped structure (Figure 6A), whereas automodified PARP1^Y986S^ has indeterminate shape and a much smaller size and PAR (Figure 6C). PARylated molecules of PARP1^Y986H^ synthesizing hyperbranched PAR look like small sphere-like structures with highly packed polymer chains (Figure 6B). We did not observe noticeable features of the morphology between autoPARylated PARP1^Y986S^ and PARP1^G972R^, the shape and size of these molecules were similar (Figure 6C and Supplementary Figure S3). Thus, the synthesis of the highly branched PAR by PARP1^Y986H^ gives rise to PARylated molecules with compact globular structure. This finding suggests that these PARP1 point mutants and PARP1 wt produce clearly distinct types of PARylated molecules in solution.

**FIGURE 7:**
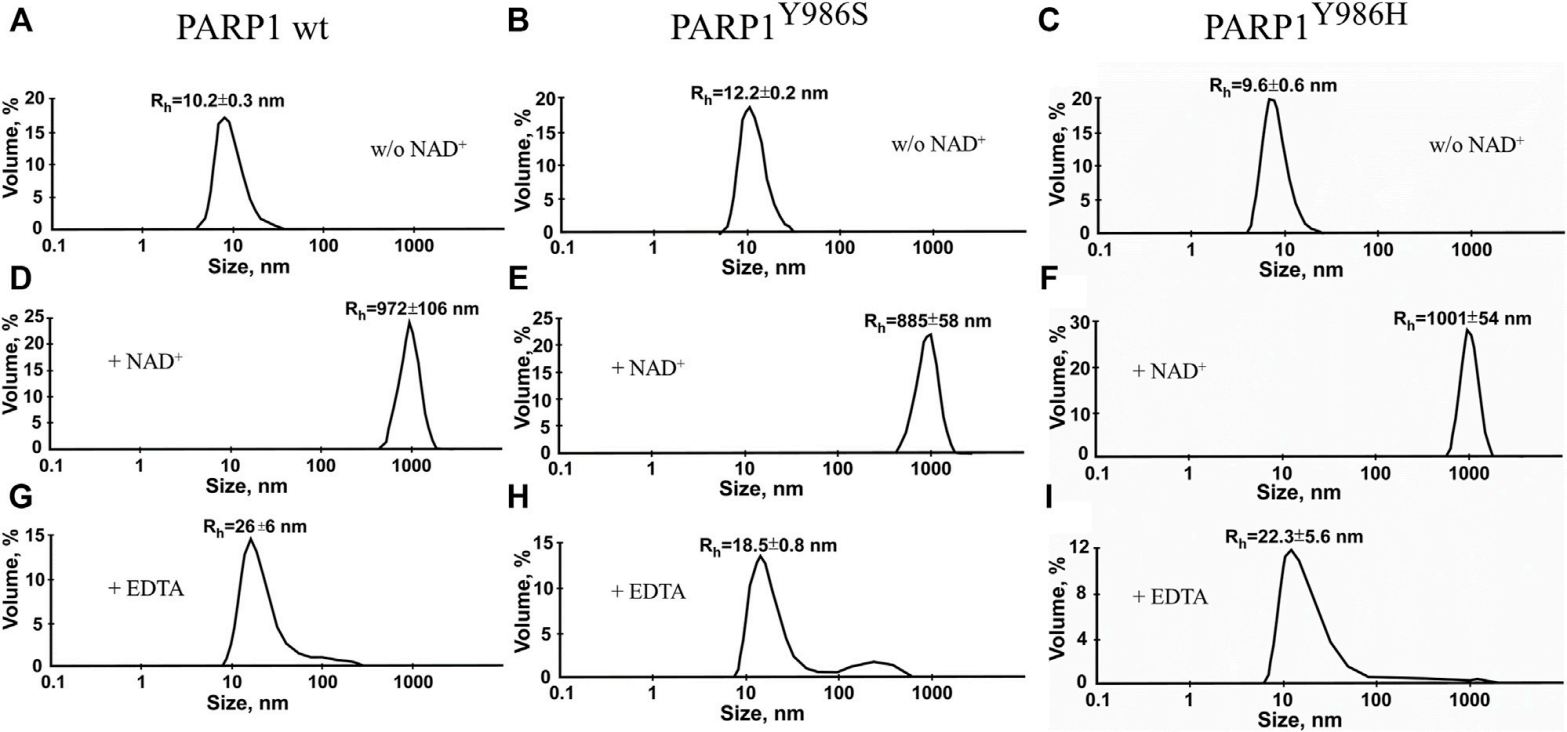
Volume-weighted size distributions of PARylated PARP1 wt and its mutants as determined by DLS. R_h_ values were measured for individual proteins **(A)** immediately after 40-min incubation with NAD^+^ and DNA **(B)** or after EDTA addition intended to disrupt aggregates of PARylated proteins stabilized with Mg^2+^ **(C).** The reaction mixtures contained 2.5 μM PARP1 or its mutant, 2.5 μM DNA (Nick), 10 mM MgCl_2_ and 1 mM NAD^+^ (where indicated). Aggregation of autoPARylated PARP1 during PAR synthesis is mediated by Mg^2+^ ions, which stabilize intermolecular contact between adjacent PARylated molecules (Vasil’eva et al., 2019). To disrupt automodified PARP1 assemblies stabilized by Mg^2+^ **(B)** EDTA (to a final concentration of 10 mM) was added to detect the real size of PARylated molecules **(C)**. R_h_ values are the mean (±SD) of three independent experiments.

We also estimated the size of auto-PARylated proteins by DLS measurements of Rh of modified PARP1 wt and its mutants (PARP1^Y986S^ and PARP1^Y986H^) (Figure 7). Under our reaction conditions, DLS measurements showed R_h_ values of ∼10 nm for PARP1 and its mutants before the initiation of PARylation (Figure 7A). The activation of PARP1 wt and its mutants as detected by DLS enabled us to measure the average R_h_ values of the PARylated proteins after the incubation with DNA and NAD^+^ (Figure 7C). PARylation of proteins expanded their size and accordingly increased R_h_ (10–18–26 nm; Figures 7A,C). For example, R_h_ of ∼26 nm was registered for auto-PARylated PARP1 wt, and R_h_ of 22.6 and 18.5 nm for automodified mutants PARP1^Y986H^ and PARP1^Y986S^, respectively (Figure 7C). Thus, the R_h_ values of PARylated proteins were substantially higher than the respective value measured for proteins before the initiation of PAR synthesis (Figures 7A,C). In addition, the increase in R_h_ values of PARylated proteins shows different extent for PARP1 wt and its mutants. For mutants, 1.5- and 2.3-fold increase of R_h_ values was observed upon PARylation of PARP1^Y986S^ and PARP1^Y986H^, respectively, whereas 2.6-fold increase of R_h_ values was detected after PARylation of PARP1 wt, that is consistent with the synthesis of shorter PAR chain by the mutants. Thus, both AFM images and DLS measurements indicate morphological differences between the wild type and mutant PARylated PARP1 molecules.

**FIGURE 8:**
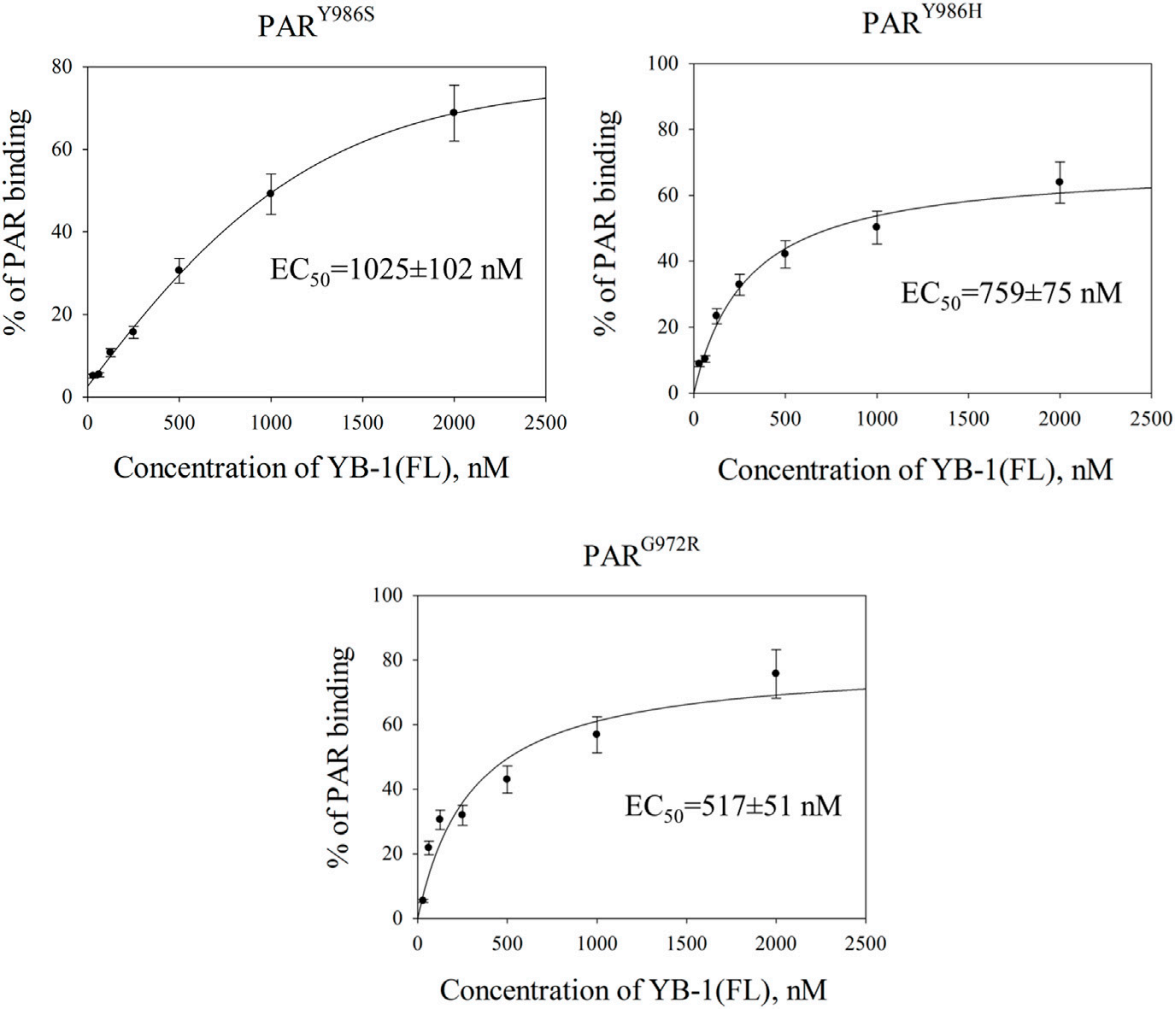
YB-1 affinity for protein-free PAR polymers synthesized by PARP1 mutants. Graphs show quantification of EMSA data (Supplementary Figure S4) and represent the mean values of three independent experiments with error bars (±SD). The binding parameters (EC50) of YB-1 interaction with PAR was determined as YB-1 concentration resulting in 50% substrate binding. EC_50_ values are the mean (±SD) of three independent experiments. The reaction mixtures contained 60 nM [^32^P]-labeled PAR and YB-1 (FL) at the indicated concentrations.

The variation of PAR structure and PARylated protein morphology may have an influence on the efficiency of YB-1 binding to PAR formed through PARP1 automodification and on the effects of YB-1 on PARP1 activity. To test this hypothesis, first we employed an EMSA to compare YB-1 binding to protein-free PARs produced by PARP1^Y986S^ (PAR^Y986S^), PARP1^Y986H^ (PAR^Y986H^), and PARP1^G972R^ (PAR^G972R^); Figure 8; Supplementary Figure S2. In contrast to PARP1 wt generating long PAR polymers with regular branching, mutants PARP1^Y986S^, PARP1^G972R^, and PARP1^Y986H^ were shown to produce shorter PAR polymers; besides, PARP1^G972R^ and PARP1^Y986H^ synthesize hypo- or hyperbranched PAR, respectively (Rolli et al., 1997; Aberle et al., 2020).

The EC_50_ values of YB-1(FL)–PAR complexes were estimated from the EMSA data obtained at various YB-1(FL) concentrations and a fixed PAR concentration (Figure 8; Supplementary Figure S4). For PAR produced by PARP1 mutants, EC_50_ values varied from 1025 to 517 nM and were substantial higher in the case of PAR^Y986S^ and PAR^Y986H^ polymers than the values for the PAR synthesized by PARP1 wt (∼564 nM; Figures 2, 8). Thus, YB-1 binds with higher affinity to long regularly branched PAR than to the short regularly branched (PAR^Y986S^) or short hyperbranched (PAR^Y986H^) polymer but has comparable binding-affinities for the PAR produced by PARP1 wt and short hypobranched PAR^G972R^ (Figures 2, 8).

These results meant that YB-1 can bind different protein-free PAR molecules; at the same time, PAR structure, namely length and branching frequency, clearly affect YB-1’s PAR-binding efficiency.

### YB-1 Stimulates the Activity of PARP1 Mutants Synthesizing Highly Branched or Short PAR Polymers

**FIGURE 9:**
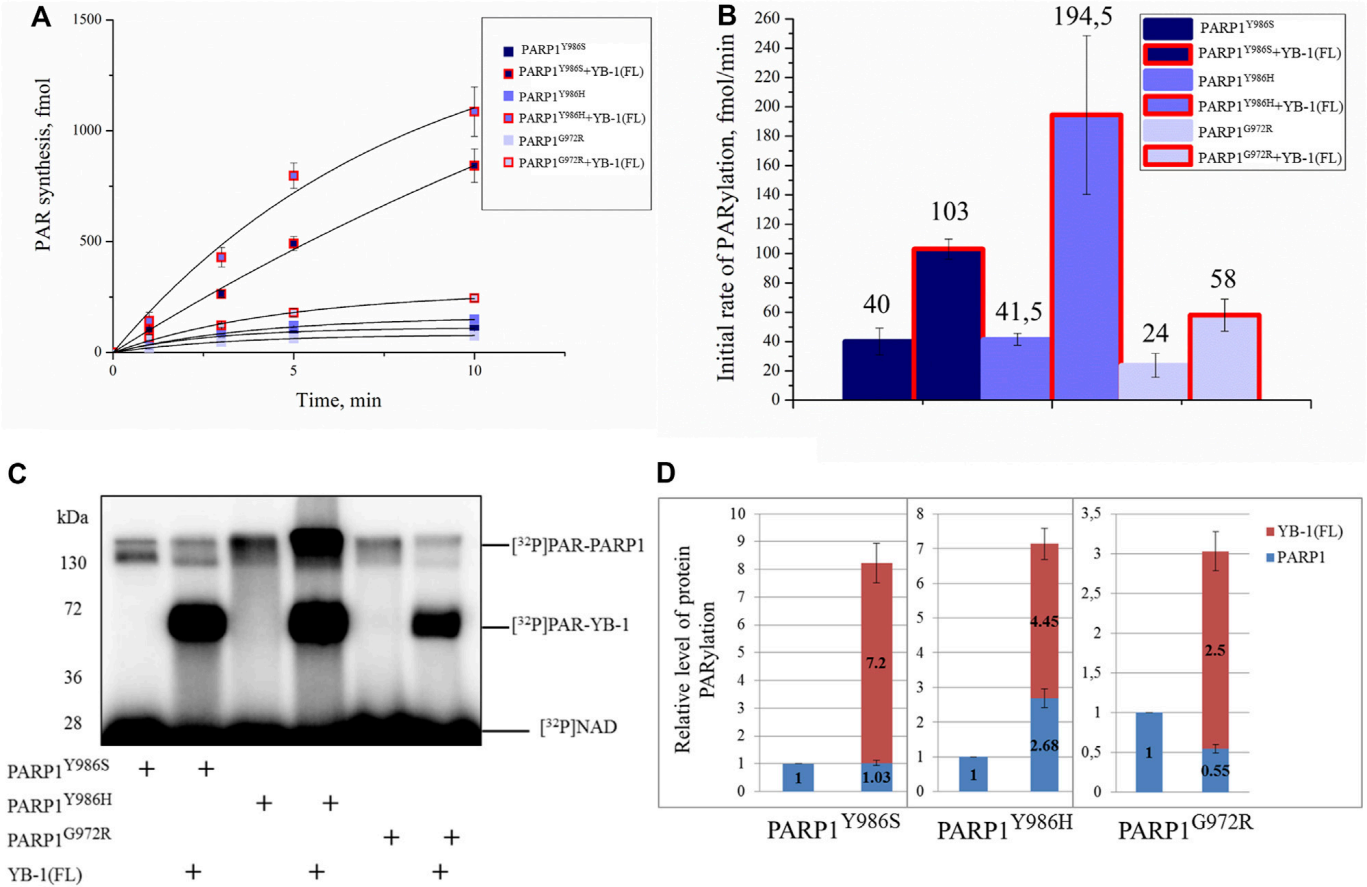
YB-1 stimulates the activity of PARP1 point mutants. **(A)** The kinetics of PAR synthesis catalyzed by PARP1 in the absence or presence of YB-1(FL) or its deletion mutants. Graphs represent the mean values of three independent experiments with error bars representing ± SD. **(B)** Initial rates of PAR synthesis estimated in the presence of YB-1(FL). PARP1 mutants at 100 nM were incubated with 0.5 OD_260_/ml DNA_act_, 20 μM NAD^+^, and [^32^P]NAD (0.4 μCi) in the presence of 2.0 µM YB-1 as indicated. [^32^P]PAR-modified proteins were precipitated with TCA and counted. The initial rates (fmol/min) were determined from a direct analysis of progressive curve **(A)** which yields P_max_ and k (Equations 1 and 3), and represent the mean ± SD of three independent measurements. **(C)** Auto-PARylation of PARP1 mutants and YB-1 trans-PARylation according to SDS-PAGE and phosphorimaging. The reaction mixtures consisted of 100 nM PARP1, 2.0 µM YB-1, 20 μM NAD^+^ [^32^P]NAD (0.4 μCi), and 0.5 OD_260_/ml DNA_act_. **(D)** The diagrams present relative magnitude of PARP1 auto-PARylation and of YB-1 trans-PARylation (the mean of three independent experiments). The relative levels of PARylated proteins were normalized to the autoPARylated PARP1 mutant alone.

Next, we investigated what happens to these differences in YB-1 binding to short, long, and hypo- and hyperbranched polymers when PAR is covalently attached to PARP1, namely, how YB-1 could influence activity of PARP1 mutants producing different types of PAR and having clear differences in morphological structure of automodified molecules (Figures 6, 7C). To address this question, we tested these PARP1 mutants’ activities in the presence of YB-1(FL) (Figure 9). Under our reaction conditions in the PARP1 wt activity assay, the overall rate of PAR synthesis catalyzed by these mutants was lower than that of the wt enzyme (Figure 9A). The addition of YB-1 stimulated the activity of PARP1 mutants thereby increasing the rate of PAR synthesis by 2.5-, 4.5-, or 2.5-fold for PARP1^Y986S^, PARP1^G972R^ and PARP1^Y986H^, respectively (Figure 9B). The data also revealed that trans-PARylation of YB-1 occurred with all PARP1 mutants (Figure 9C). Of note, in the case of PARP1^Y986S^ and PARP1^G972R^, the level of YB-1 PARylation was significantly higher than the level of PARP1 auto-PARylation, reaching up to 87% of total protein modification (Figure 9D). One could say that PAR–YB-1 interactions are influenced by the structure of PAR attached to PARP1 (its length and frequency of branching), which affects the strength of stimulation of overall PAR synthesis and the ratio of YB-1 trans-PARylation to PARP1 auto-PARylation. Indeed, with mutants PARP1^Y986S^ and PARP1^G972R^ producing short PAR, we observed a YB-1–dependent increase in the PAR synthesis rate—that was similar to that seen with PARP1 wt—but a much higher level of YB-1 trans-PARylation (83 and 87%, respectively). With the PARP1^Y986H^ mutant producing hyperbranched PAR, YB-1 had a moderate effect on the rate of PAR synthesis, and the level of its trans-PARylation was comparable with that observed in the reaction catalyzed by PARP1 wt (∼60%; Figure 5D). Thus, PAR structure represents an important determinant for the stimulation of PARP1 activity with YB-1, because this parameter determines the level of YB-1 trans-PARylation, which plays a key role in the stimulation of PARP1 activity by increasing the overall PAR product yield.

## Discussion

YB-1 has emerged as a key regulator of cell metabolism and performs diverse biological functions, including modulation of gene transcription, mRNA translation, chromatin modification, cell proliferation, and a stress response (Mordovkina et al., 2020; Sangermano et al., 2020). Moreover, YB-1 protein expression is upregulated in human cancers including breast, prostate, and ovarian cancers and melanoma and can correlate with aggressive tumor cell phenotypes and tumor formation and progression (Sangermano et al., 2020). YB-1 is thought to be a multifunctional protein capable of binding to single- or double-stranded DNA or RNA and can interact with proteins taking part in various metabolic pathways including gene transcription, mRNA translation, DNA repair, and control of cell cycle progression parameters (Skabkin et al., 2006). Our recent data show that aside from these functions, YB-1 can be involved in the regulation of activity of PARP1, which is a key player in DNA repair (Alemasova et al., 2018; Naumenko et al., 2020). PARP1 acts primarily as a sensor of DNA strand breaks and forms DNA repair foci *via* local synthesis of PAR at DNA damage sites (Satoh and Lindahl, 1992; Caldecott, 2014). To date, approximately 1500 proteins have been identified as acceptors of the covalent PARylation performed by PARP1, and many of these proteins are also PAR-binding factors, so-called “readers” of PAR (Tanabe et al., 2015; Ayyappan et al., 2021). Accordingly, PARP1 activity appears to be regulated by proteins both *via* the assembly of protein–protein complexes on damaged DNA and/or by PAR binding that results in trans-PARylation of target proteins, a decrease or increase in the magnitude of PARP1 auto-PARylation, and a switch of PARP1 specificity from auto-PARylation to trans-PARylation (Alemasova and Lavrik., 2019). This notion suggests that other proteins can regulate the “PAR-code” including PARP1 auto-PARylation and protein trans-PARylation patterns and PAR polymer structure (Luo and Kraus, 2012; Alemasova and Lavrik., 2019; Reber et al., 2021). A number of proteins that cooperate with PARP1 and are targeted by PARylation contain a highly basic intrinsically disordered protein region, implying an important role of such a region in PAR binding (Fischbach et al., 2018; Singatulina et al., 2019; Krüger et al., 2019; Rank, 2019). Among them, more than 100 RNA-binding proteins have considerable PAR-binding affinity and are PARylated in a cellular context (Gagne et al., 2008; Jungmichel et al., 2013; Zhang et al., 2013; Daniels et al., 2014). So far, only a few proteins have been shown to regulate the PARP1 activity and PAR-dependent processes *in vitro* and *in vivo* (Krietsch et al., 2012; Altmeyer et al., 2015; Sun et al., 2016; Singatulina et al., 2019). Expectedly, the regulation of PARP1 activity by RNA-binding proteins is being actively investigated (Bock et al., 2015; Singatulina et al., 2019; Leung 2020). RNA-binding proteins usually contain an RNA recognition motif (RRM), RG/RGG repeats, and serine/arginine-rich (SR), lysine-and-arginine-rich (KR), and/or arginine/glycine-rich (RG/RGG) repeats (Corley et al., 2020); all these regions possess both RNA- and PAR-binding properties (Teloni and Altmeyer, 2015). Herein, we report that the CTD of YB-1 plays a critical role in the binding of YB-1 to PAR and is required for YB-1–driven regulation of PARP1 activity. The CTD is the largest region of YB-1 and mediates protein–protein and protein–nucleic acid interactions and is essential for the function of this protein *in vivo* (Mordovkina et al., 2020), but the participation of the CTD in the regulation of PARP1-related reactions is poorly understood. The CTD has a disordered structure and predominantly contains clusters of basic amino acid residues, which are followed by clusters of acidic residues termed a charged zipper (Figure 1). On the basis of our previous findings, the regulation of PARP1 by YB-1 was expected to depend on the formation of two types of complexes mediated by YB-1 interaction with DNA in a ternary complex with PARP1 and by an interaction with PAR during PARP1 auto-PARylation (Naumenko et al., 2020). We also supposed that the YB-1 CTD plays a central role in the regulation of PARylation reactions because its deletion inhibits YB-1–dependent stimulation of PAR synthesis and YB-1 trans-PARylation (Alemasova et al., 2018; Naumenko et al., 2020). Assuming that the CTD is a key module with regards to PAR binding, a direct participation of clusters of basic amino acid residues from the YB-1 CTD in PAR recognition was hypothesized in the present work. Here we were able to show that clusters of positively charged residues within the CTD are necessary for YB-1 interaction with PAR. Our results revealed that the reduced PAR-binding activity of YB-1 mutants (featuring a deletion of two positively charged clusters in the CTD) correlates well with diminished trans-PARylation (Figures 2, 5). Thus, the noncovalent YB-1–PAR interaction and covalent YB-1 trans-PARylation are tightly linked within these processes, and the CTD may be regarded as a key module for PAR binding and regulation of PARP1 auto-PARylation by YB-1. We also found that YB-1 stimulates the activities of mutant PARP1 proteins that produce short branched PAR (i.e., PARP1^Y986S^), short hyperbranched PAR (PARP1^Y986H^), or short hypobranched PAR (i.e., PARP1^G972R^) (Figure 9), implying that YB-1 modulates PARP1 activity in the context of diverse PAR structures. At the same time, the size (i.e., number of ADP-ribose units) and shape (i.e., branching frequency) of protein-free PAR affect YB-1 binding affinity for the polymers, and these PAR characteristics greatly influence the ratio of PARP1 auto-PARylation to trans-PARylation of YB-1 in case of PARP1 mutants. For example, PARP1^Y986S^ and PARP1^G972R^ produce short-chain PAR and predominantly catalyze YB-1 trans-PARylation.

It should be noted that the level of YB-1 trans-PARylation is only moderately affected by the type of damaged DNA structure (Naumenko et al., 2020), whereas the PAR structure affects the trans-modification of this protein (Figure 9).

**FIGURE 10:**
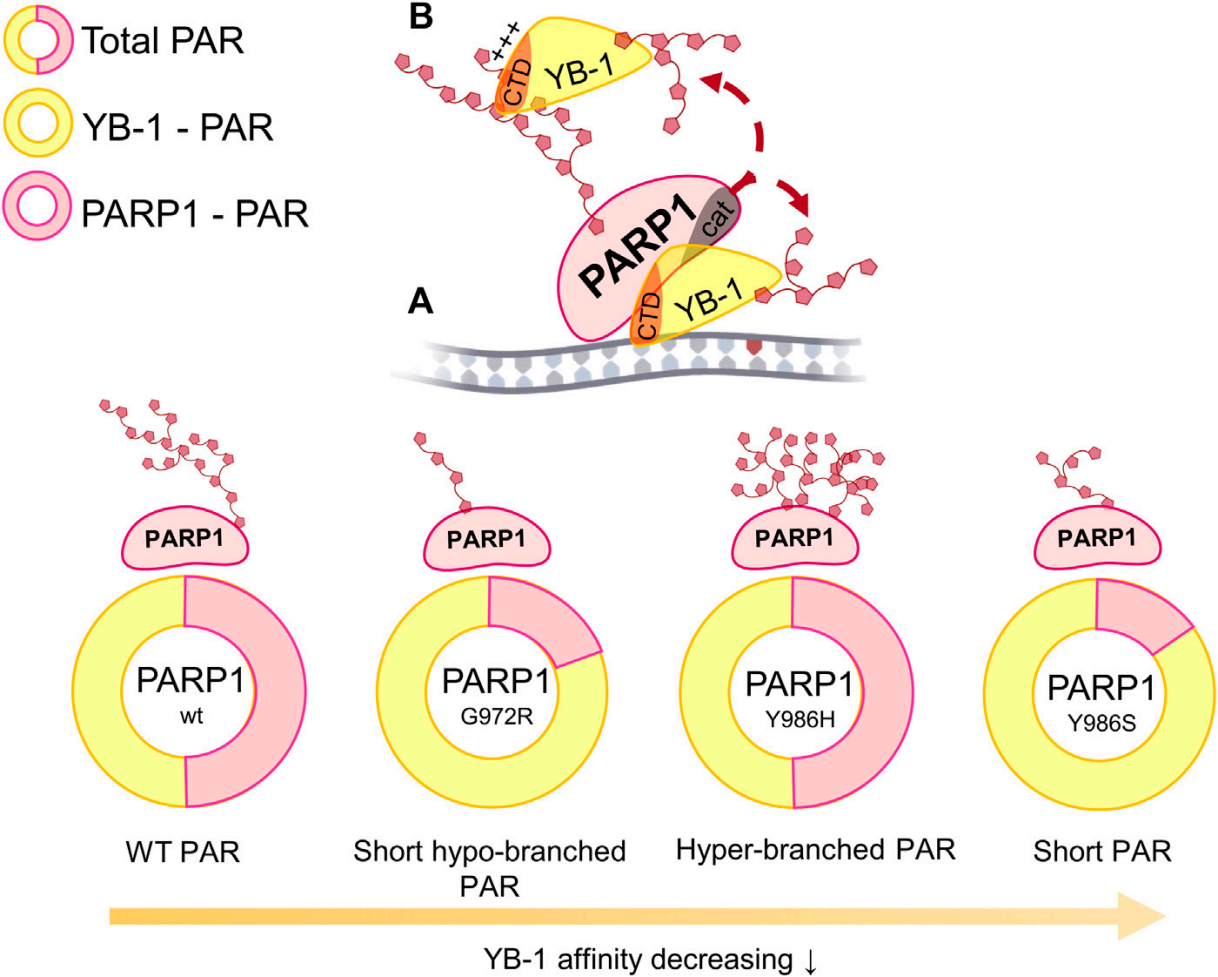
The influence of various lengths and branching frequencies of the PAR synthesized by PARP1 point mutants (Y986H, Y986S, and G972R) on the YB-1 affinity to PAR, the magnitude of PARP1 auto-PARylation (highlighted in rosy) and YB-1 trans-PARylation (highlighted in yellow) during the stimulation of PARP1 activity in ternary complex “PARP1–YB-1–damaged DNA” **(A)** as well as during the interaction of YB-1 with PAR *via* its C-terminal domain (CTD; **(B)**.

To sum up the current results, we propose a model where the PAR structure formed during PARP1 auto-PARylation plays an important role in YB-1–PAR complex formation, YB-1 trans-PARylation, and PARP1 auto-PARylation (Figure 10). For instance, PAR structure and the YB-1 interaction with PAR should contribute primarily to the regulation of YB-1 trans-PARylation by changing the efficiency of YB-1 trans-modification and hence the stimulation of PAR synthesis by an exchange between PARylated and non-PARylated YB-1 molecules, in line with the model suggested by us earlier (Naumenko et al., 2020). This notion suggests that YB-1 may be viewed as “PAR-code” regulating the factors that interact with PAR and thereby influences PARP1 activity (Reber and Mangerich, 2021). The observed patterns of PAR synthesis may be extrapolated to PARP1’s protein partners containing PAR-binding disordered regions with clusters of positively charged amino acid residues.

## Data Availability

The original contributions presented in the study are included in the article/Supplementary Material, further inquiries can be directed to the corresponding author.
