# Supplementary material for: The C-Terminal Domain of Y-Box Binding Protein 1 Exhibits Structure-Specific Binding to Poly(ADP-Ribose), Which Regulates PARP1 Activity
Source: Front Cell Dev Biol. 2022 Jun 21;10:831741. doi: 10.3389/fcell.2022.831741 (PMC9253770; doi:10.3389/fcell.2022.831741)
Supplement: Supplementary file 1 [file DataSheet1.pdf]

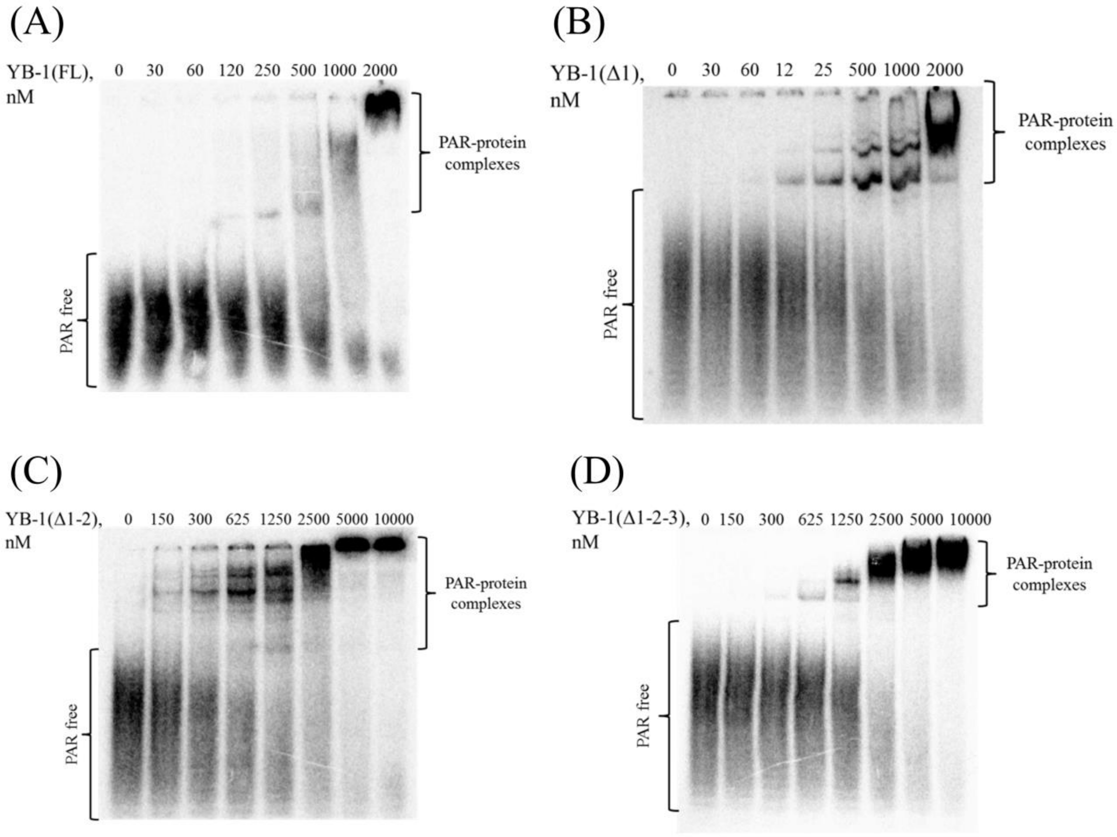

Supplementary Figure 1. EMSA data on the binding of YB-1 or its mutants to PAR. The reactions were carried out as described in Material and Method sections. Nondenaturing PAGE in a 5% gel (acrylamide/bis-acrylamide at 37.5:1) was performed for the analysis of complexes PAR–YB-1(FL) (**A**) and PAR-YB-1(Δ1) (**B**), 10% PAGE (acrylamide/bis-acrylamide at 75:1) for the analysis of complexes PAR–YB-1(Δ1-2) (**C**) and PAR–YB-1(Δ1-2-3) (**D**) in TBE buffer at 4°C followed by phosphorimaging on a Typhoon FLA 9500 Biomolecular Imager (GE Healthcare). Bound- and unbound-PAR signals were quantified in the Quantity One Basic software. The data were fitted to an equation using the SigmaPlot software.

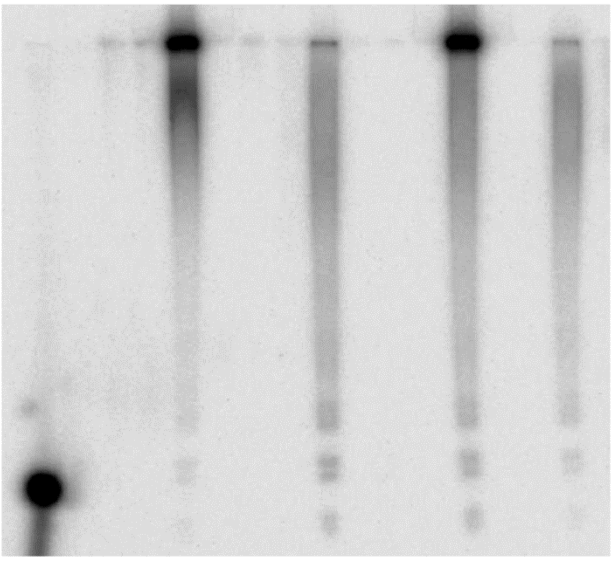

Supplementary Figure 2. Size distribution of protein-free poly(ADP-ribose) [PAR] synthesized by PARP1 wt and mutants. [^{32}P]-labeled PAR was synthesized as described in Materials and Methods. PAR samples produced by PARP1 wt and PARP1 point mutants were analyzed by electrophoresis in a denaturing urea 20% polyacrylamide gel with subsequent phosphorimaging. [^{32}P]-labeled–PAR concentration was estimated as the amount of monomeric ADP-ribose incorporated into a polymer. [^{32}P]NAD$^+$ signal intensity (arbitrary units, a.u.) was used as a standard. The amounts of [^{32}P]PAR produced were calculated by acquiring the signals from PAR resolved by the urea PAGE.

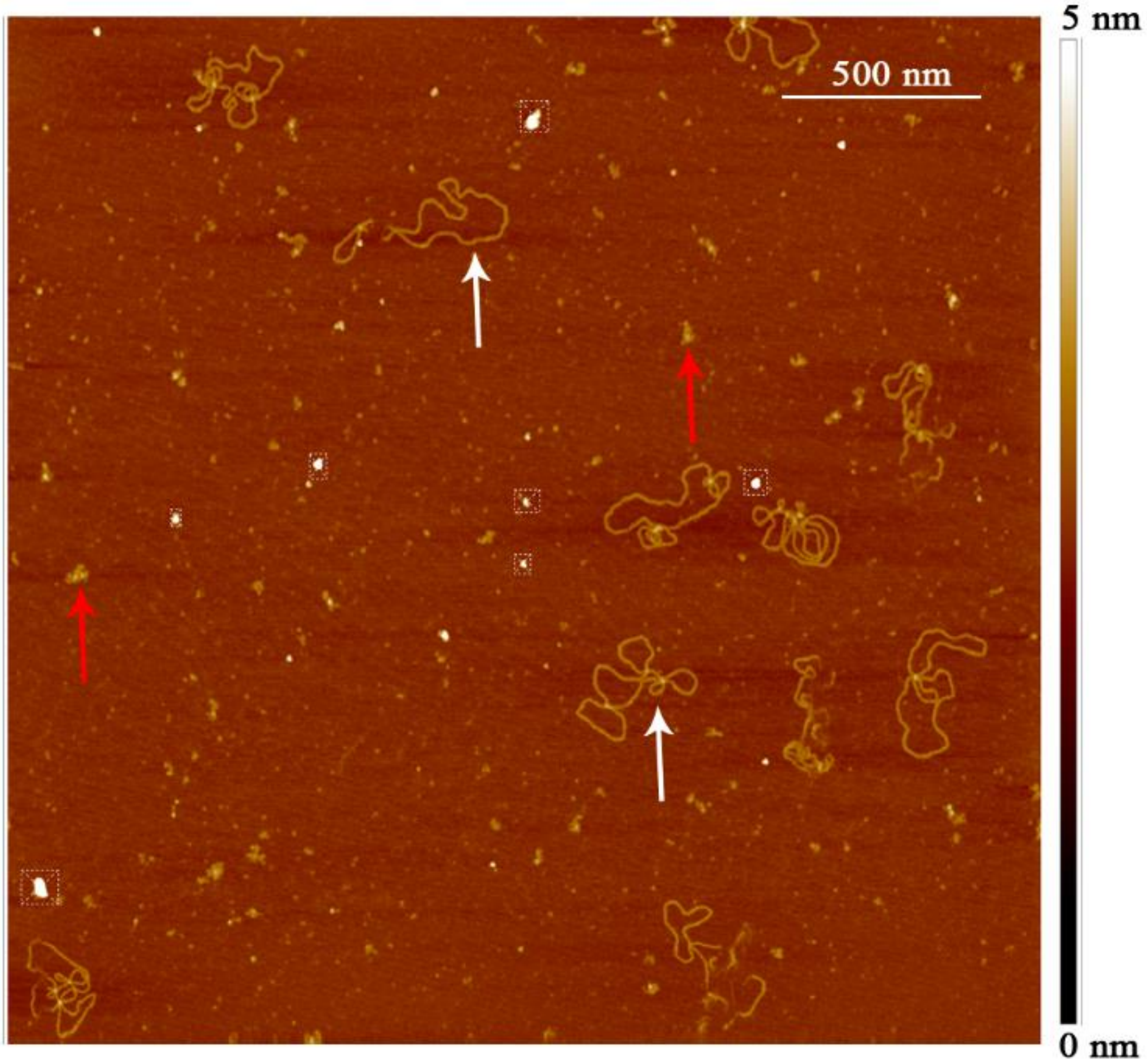

Supplementary Figure 3. AFM visualization of the shape of PAR polymers synthesized by PARP1^{G972R}. The large scale of AFM image illustrates auto-PARylation of PARP1^{G972R}. White arrows indicate plasmid DNA molecules, and red arrows point to PARylated proteins. Scale bar: 500 nm; Z scale: 5 nm.

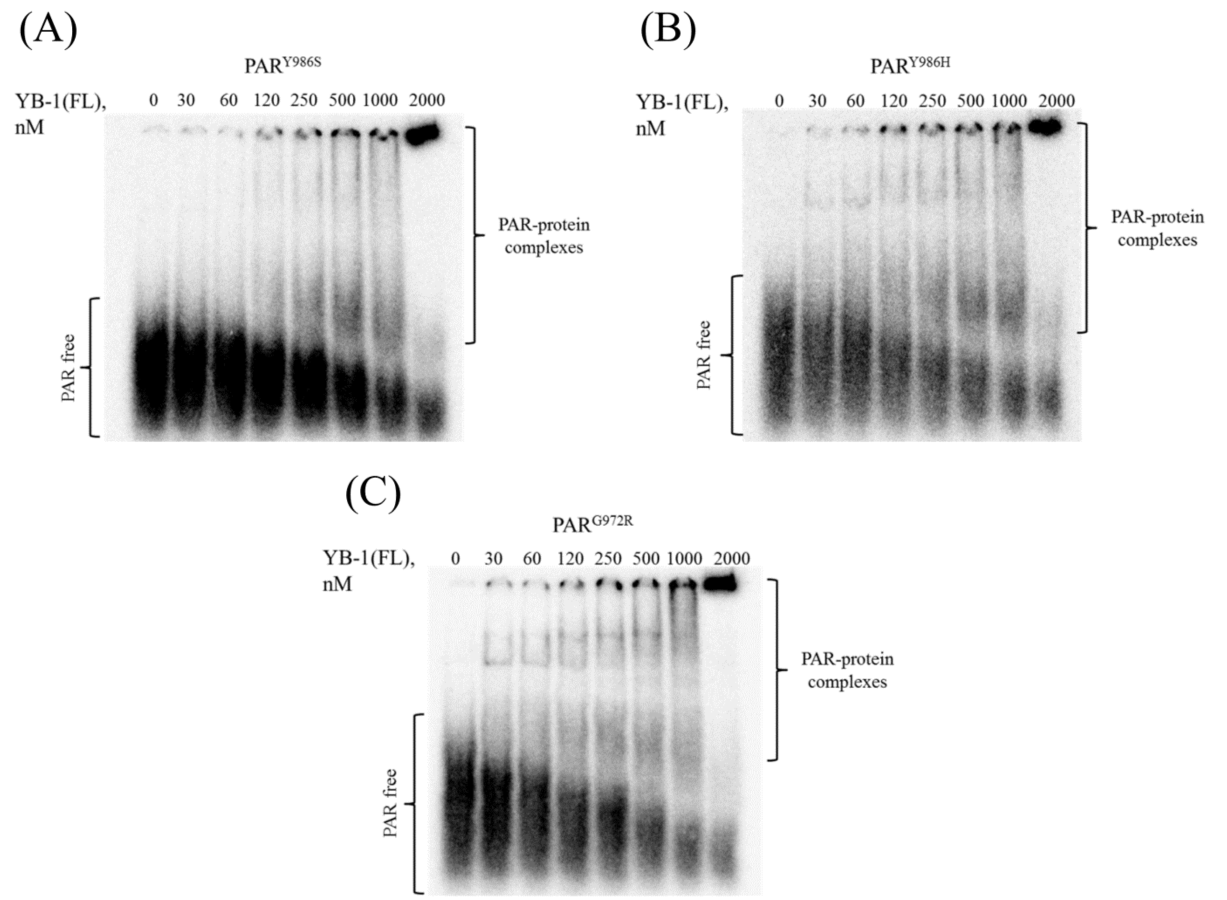

Supplementary Figure 4. EMSA data for YB-1(FL) binding to protein-free PAR produced by PARP1^{Y986S} (**A**), PARP1^{Y986H} (**B**) and PARP1^{G972R} (**C**). The reactions were carried out as described in Material and Method section. Nondenaturing PAGE in 10% PAGE (acrylamide/bis-acrylamide at 75:1) for the analysis of complexes PAR^{Y986S}–YB-1(FL), PAR^{Y986H}–YB-1(FL), and PAR^{G972R}–YB-1(FL) in TBE buffer at 4°C followed by phosphorimaging on a Typhoon FLA 9500 Biomolecular Imager (GE Healthcare). Bound- and unbound-PAR signals were quantified in the Quantity One Basic software. The data were fitted to an equation using the SigmaPlot software.
